# The Eph/Ephrin system in primary bone tumor and bone cancer pain

**DOI:** 10.18632/aging.204852

**Published:** 2023-07-06

**Authors:** Lujuan Wang, Wei Li, Yong Pan

**Affiliations:** 1 Hunan Cancer Hospital and The Affiliated Cancer Hospital of Xiangya School of Medicine, Central South University, Changsha 410013, Hunan, China; 2 Hunan Key Laboratory of Tumor Models and Individualized Medicine, The Second Xiangya Hospital of Central South University, Changsha 410011, Hunan, China

**Keywords:** primary bone tumor, Eph/Ephrin

## Abstract

The family of Eph receptor tyrosine kinases and their Ephrin ligands system constitutes a bidirectional signaling pathway. Eph/Ephrin system coordinate a wide spectrum of pathologic processes during development, metastasis, prognosis, drug resistance and angiogenesis in carcinogenesis. Chemotherapy, surgery and radiotherapy are the most commonly used clinical treatments for primary bone tumors. Therefore, surgical resection is often unable to completely eliminate the tumor, and this is the main cause of metastasis and postoperative recurrence. A growing body of literature has been published lately revitalizing our scientific interest towards the role of Eph/Ephrins in pathogenesis and the treatment of bone tumor and bone cancer pain. This study mainly reviewed the roles of Eph/Ephrin system that has both tumor-suppressing and -promoting roles in primary bone tumors and bone cancer pain. Understanding the intracellular mechanisms of Eph/Ephrin system in tumorigenesis and metastasis of bone tumors might provide a foundation for the development of Eph/Ephrin targeted anti-cancer therapy.

## INTRODUCTION

The erythropoietin hepatocyte kinase receptor (Eph) and its ligand (Ephrin) belong to the largest family of receptor tyrosine kinases (RTKs), which have complex functions in living organisms. Equally named for its expression in an erythropoietin-producing human hepatocellular carcinoma cell line, Eph is known to function in many types of cells signaling. Hirai first identified and demonstrated in 1987 that the product of Eph gene expression was a transmembrane protein [1], which was then thought to be a receptor for growth factors and was hypothesized to play a role in tumorigenesis. Following continuous in-depth research, the effect of Eph/Ephrin in the development and progression of cancer has been well established [2]. Eph/Ephrin is also involved in cytoskeleton formation, cell adhesion, intercellular junctions, cell morphology and motility through bidirectional signaling, affecting neuronal development and skeletal homeostasis, and is a key factor in angiogenesis and lymphangiogenesis.

Primary bone tumors, notably sarcomas, affect youngsters the most since they originate from the osteoblasts responsible for bone growth. Chemotherapy, surgery and radiotherapy are the most commonly used clinical treatments. However, surgical resection often does not completely eradicate the tumor, which is the main cause of metastasis and postoperative recurrence, leading to a high mortality rate. In Addition, bone tumors frequently penetrate a large number of skeletal areas, rendering them unable to repair themselves and compromising the patient’s quality of life. As a consequence, treating bone tumors and regenerating bone in the clinical setting is difficult. The lack of approved treatments has led to research on various alternative treatments in recent decades [3]. Several studies have reported that Eph/Ephrin works in osteogenesis, osteoclast signaling and maintenance of bone homeostasis and fracture healing, such as EphB4/EphrinB2 reverse signaling inhibits osteoclast differentiation and positive signaling enhances osteoclast differentiation, and through this bidirectional signaling is involved in bone homeostasis and bone reconstruction [4–6]. It has been demonstrated that the Eph/Ephrin system is involved in the regulation of inflammatory and neuropathic pain. For example, in the rat BCP model, EphB1/EphrinB1 may be in the maintenance of mechanical nociception by regulating the expression of inflammatory cytokines in the spinal cord [7]. This study focuses on the mechanism of action of Eph/Ephrin in primary bone tumors and bone cancer pain (BCP) caused by metastatic bone tumors, providing new ideas for the study of bone tumors and BCP clinical targeting drugs.

## Structure of eph/ephrins and their bidirectional signals

**Figure 1:**
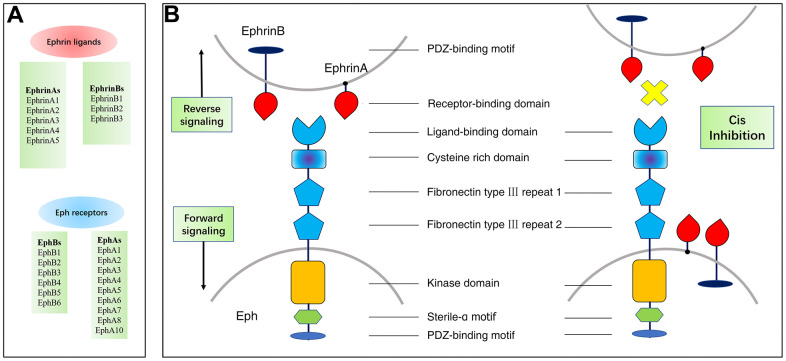
**Members and structure of Eph/Ephrins and their bidirectional signals.** (**A**) Members and Structure of Eph/Ephrins. (**B**) Both Eph receptors and ephrin are expressed in opposing cells and interact in trans to activate forward/reverse signaling. Eph receptors and ephrin are co-expressed in the same cell and interact in cis. Cis inhibition has been shown to inhibit trans signaling.

Tyrosine kinase receptors (RTK) are cell surface transmembrane proteins that play a critical function in the transduction of extracellular signals to the intracellular compartment. The erythropoietin-producing hepatocyte (Eph) receptor is the largest member of the tyrosine protein kinase receptor family and its ligand is mainly expressed on the surface of the cell and is named Ephrin (Eph receptor interacting protein). Based on sequence homology and binding specificity, there are two classes of Eph receptors: class EphA, which comprises nine members (EphA1-A8 and EphA10), and subclass EphB, of which five members (EphB1-B4 and EphB6) are included. Eph receptors are triggered upon ligand Ephrins-binding to the interacting ligands. There are eight types of ephrin ligands, which are also divided into two categories: EphrinAs (A1-A5), which are partially attached to the cell membrane via glycosylphosphatidylinositol (GPI), and EphrinBs (B1-B3), which span the cell membrane and possess a cytoplasmic signaling domain (Figure 1).

Eph-Ephrin signaling is unique in that it is bidirectional. It is known as forward signaling in cells expressing Eph receptors or reverse signaling in cells expressing Ephrin ligands. With a few exceptions, EphA receptor-mixed binding of EphrinAs ligands, EphB receptor-mixed binding of EphrinBs ligands [8]. Ephrin activates Eph most commonly through cell-to-cell interactions, where the Eph receptor interacts with its Ephrin ligand and then heterodimerizes, subsequently forming a tetrameric complex that leads to receptor tyrosine phosphorylation and kinase activation. Cellular release of Ephrin-As has been reported to activate the EphA receptor remotely. In addition, Eph receptors can be stimulated by other surface cell receptors (e.g. EGFR) [9]. EphrinA3 and EphrinB3 can bind to acetyl heparan sulfate proteoglycans, thereby enhancing Eph receptor of the activation and signals transmission [10, 11].

The Eph receptor is a type I transmembrane protein with a normally conserved structure. The ligand-binding domain, the cysteine-rich region and two type III fibronectin repeats comprise the extracellular domain of the receptor, while the intracellular region consists of the near-membrane domain, the protein tyrosine kinase (Pkinase-Tyr) domain, the sterile alpha constitutive motif (SAM) and the C-terminal PDZ-binding motif [8, 12]. Ephrin ligands are typically memory-linked, and Ephrin-A ligands are fixed to the membrane via a glycophosphatidylinositol, in contrast to Ephrin-B ligands with transmembrane structural domains and cytoplasmic regions with PDZ structural domains [13]. Eph receptor binding to Ephrin ligand results in tyrosine phosphorylation of the cytoplasmic tail of Ephrin-B ligand and can interact with signaling molecules containing the SRC-homologue-2 structural domain [14].

Eph/Ephrins signaling plays an important role in many biological processes that contribute to development and homeostasis *in vivo*. They regulate cell morphology, adhesion, motility, proliferation, survival, and differentiation by mediating contact-dependent communication between the same or different cell types [14]. During the course of adult organisms, the Eph/Ephrin signaling system provides regulation of synaptic remodeling, epithelial differentiation, bone remodeling, immunity, insulin secretion, and stem cell self-renewal [15, 16]. As well as being expressed in normal tissues, Eph receptors are also expressed in tumor cells and the tumor microenvironment and perform important processes associated with tumorigenesis and tumor metastasis [17]. However, Eph receptor expression is not always add up in in tumors, and the expression of certain Eph molecules is downregulated in many malignancies, suggesting that Eph receptors can both promote tumor development and act as suppressors to inhibit tumor progression [12]. Eph/Ephrin signaling has been shown to participate in the predevelopment of many tumors, including melanoma, neuroblastoma, malignant glioma, pancreatic cancer, colon cancer, lung cancer, gastrointestinal tumors, esophageal cancer, liver cancer, etc. Eph/Ephrin signaling promotes epithelial mesenchymal transition and invasion of many tumors by activating Src, STAT3, MMP8 and RAC1 [18–21].

## Eph/Ephrin and primary bone tumors

### Eph/Ephrin and myeloma

Myeloma originates from the hematopoietic tissue of bone marrow and is a malignant tumor caused by excessive proliferation of plasma cells. Myeloma is prone to multiple bone damage, also known as multiple myeloma (MM), and accounts for about 10% of hematologic malignancies [22]. Prognosis is relatively difficult in patients with MM who have bone pain, pathological fractures, vertebral collapse and hypercalcemia in approximately 80% of patients [22]. EphB4/EphrinB2 signaling take an essential leverage at osteogenic and osteoclastic signaling and functional maintenance [23]. Pennisi et al. found reduced expression of EphrinB2/EphB4 in bone marrow mesenchymal stem cells of MM patients [24].

It was found that Wnt3a promotes the expression of the target gene EphB4 in MSCs from MM patients, and that EphrinB2 -Fc activates p-EphB4, which in turn inhibits osteogenesis in MSCs from MM patients [24]. Oshima et al. demonstrated that Osteomyeloma cells generate Fuchs-related protein 2 (sFRP-2), which is an inhibitor of the Wnt pathway which inhibits osteoblastogenesis and bone formation in MM patients [25]. Therefore, the downregulation of EphB4 in myeloma patients may be due to the inhibition of the Wnt signaling pathway in myeloma. While EphB4-Fc was found to inhibit myeloma growth, osteoclastogenesis and angiogenesis, along with stimulating osteoblastogenesis and bone formation, the treatment with EphrinB2-Fc had no effect on osteoclastogenesis and myeloma growth by stimulating angiogenesis, osteoblastogenesis and bone formation. Highly Modulated Expression of Endogenous EphB4 and EphrinB2 in Osteoblasts (e.g., treatment with Wnt3a) or exogenous increase in EphB4 levels (e.g., treatment with EphB4-Fc) may help restore bone remodeling capacity while inhibiting multiple myeloma growth, bone disease, and angiogenesis. Guan et al. found that EphA3 was highly expressed in MM [26]. Francesco et al. showed that There is an emerging view that EphA3 has a critical function in the pathogenesis of MM and provides support for the suggestion that it targets new therapeutic strategies for MM. EphA3 deficiency inhibited angiogenesis in MM patients’ vascular endothelial cells (MMECs) *in vitro*, but EphA3 deficiency did not affect the angiogenic function of normal endothelial cells (ECs). The treatment with anti-EphA3 antibody (EphA3 mAb) substantially suppressed the progression of tumor growth and angiogenesis in MM-derived mouse xenograft models *in vivo* [27]. EphA3 also affects the adhesion of MMECs cells, but has little or no effect on apoptosis. Mounting evidences have shown that CDK5 is high-overexpressed in multiple myeloma, which mediates bortezomib resistance and is also involved in the AKT pathway. Therefore, Ding et al. suggested that EphA4 interacts with and promotes CDK5 expression, then activates P-AKT to mediate multi-myeloma cell adhesion-mediated drug resistance [28]. Although the exact mechanism of its involvement in drug resistance has not yet been investigated, it may be a molecular target for drug resistance in MM patients. The interplay with bone marrow stromal cells (BMSCs) is thought to be an important mechanism of multiple myeloma (MM) cell progression, and exosomes are a key mediator of intercellular communication. Recently, it was demonstrated that Bone marrow stromal stem cell-derived exosomes miR-10a and miR-16 may participate in MM progression as well by regulatory of EphA8 or IGF1R/CCND1/CUL3/ELAVL1 [29].

### Eph/Ephrin and osteosarcoma (OS)

Osteosarcoma is a heterogeneous malignant stromal tumor. Currently, it is a cornerstone of osteosarcoma treatment that complete surgical resection, but in the case of advanced or unresectable osteosarcoma, such as metastasis, usually metastasizes to the lung, its treatment is still challenging, and osteosarcoma targeting and immunotherapy still need to be further improved. The increased expression of EphA2 receptor and its ligand EFNA1 in osteosarcoma tissue was detected using whole-genome microarray. Patients with EphA2-positive showed a trend toward inferior overall survival [30]. The high expression of EFNA1 and EphA2 may be closely related to the known carcinogenic pathway, for example, by regulating the activation of mitotic signal pathway leading to the formation and progression of osteosarcoma [31]. Ephrin expression profiling demonstrated that Ephrin-A5, Ephrin-B3 and Ephrin-B1, ligands for Eph receptors including EphB2 and EphA3, are also expressed in a vast majority of osteosarcoma specimens [31]. Using liposomes loaded with doxorubicin and modified with YSA peptide to target EphA2 can reduce the adverse effects on primary osteoblasts and maximize the toxicity to SaOS-2 osteosarcoma cells [32]. EphA2-directed CAR T cells can effectively eliminate OS and ES tumor cells that stably express EphA2 in immunodeficient mice and can effectively extends the survival of mice [33]. The combination of pazopanib and trametinib has been shown to significantly reduce EphA2 and IL-7R expression, and blocking EphA2 expression significantly reduces the proliferation and migration of osteosarcoma cells [34]. ALW-II-41-27 is a validated EphA2 inhibitor that has shown anti-neoplastic activity in different cancer types and preclinical models [35, 36], and some researchers found that the EphA2 inhibitor ALW-II-41-27 has remarkable dose-dependent antitumor effects in an *in vitro* model derived from patients with osteosarcoma [37]. EphA7 expression levels are abundant in osteosarcoma tissues, and miR-448 is able to target EphA7 to inhibit the growth and invasion of osteosarcoma cells [38]. The lncRNA HCP5 knockdown could block the expression of EphA7 by binding to miR-101 and then induce apoptosis in osteosarcoma cells [39]. Individually, Ephrin-A1, Ephrin-A4 and Ephrin-B2 were expressed in normal bone specimens, osteosarcoma tissue specimens and osteosarcoma cell lines. In contrast, Ephrin-A3, Ephrin-A5 and Ephrin-B1 were expressed only in specific osteosarcoma tissues [40, 41]. Among them, Ephrin-B1 has been shown to be expressed in both osteosarcoma cell lines and blood vessels and is associated with a poor clinical prognosis [40]. Ephrin-A4 was expressed in 82.9% of osteosarcoma cases, 58.9% of which were cytoplasmically localized. Cytoplasmic patterns were markedly correlated with aggressive histopathological type of osteosarcoma, advanced stage, presence of metastases, poorer response to neoadjuvant chemotherapy, and tended to be associated with shorter event-free survival [42].

### Eph/Ephrin and Ewing’s sarcoma

Ewing’s sarcoma (ES) is a rare and highly aggressive tumor, mostly found in the long bone stem and pelvis, accounting for 3% of childhood tumors. It is a relatively common bone tumor in childhood and adolescence, with a survival rate of 30% for patients with tumors [43]. ES is sensitive to radiotherapy but prone to metastasis, and radiotherapy alone is not effective in metastatic and recurrent cases. Although the survival rate of ES patients has improved with the current combination of treatment options, the overall prognosis of patients with metastatic tumors is poor. The role of EphA2 in tumor angiogenesis has been demonstrated [13]. Based on the biological roles of EphA2 in promoting angiogenesis, Sáinz-Jaspeado et al. [44] explored the functional role of EphA2 and its relationship with caveolin-1 (CAV1) in EWS angiogenesis. They showed that the EphA2-CAV1 axis promotes the migration of endothelial cells to the tumor, thus facilitating the angiogenesis of ES in a ligand-dependent manner. If this effect can be specifically blocked, it will slow down the tumor progression and reduce the risk of tumor metastasis, which will be helpful for clinical treatment. In addition, it was shown that EphA2 was previously associated with vascular mimicry, as an important contributor to ES malignancy and prognostic deterioration [45]. Analysis of public gene expression databases in microarrays confirmed that EphA2 was expressed at higher levels in Ewing’s sarcoma than in normal tissue [37]. The EphA2 receptor is also a crucial factor in the metastasis and recurrence of Ewing sarcoma. Studies have shown that p-EphA2^S897^ is associated with malignant invasion of ES, so a blockade of EphA2 function may be a promising therapeutic solution [46]. A transmembrane RTK encoded by EphB2 binds to ephrin ligands and mediates communication between cells through bidirectional signaling [13]. That its functional implication in promoting EwS cell aggression is supported by several arguments. The overall survival of EwS patients with tumors carrying high levels of EphB2 expression is worse than that of patients with tumors which express low-levels of the gene. Overexpression of EphB2 increases the aggressiveness of EwS2 in three-dimensional cultures. The miR-145 enabled us to identify EphB2 as a facilitator of EwS metastasis, which can serve both as a predictor of tumor behavior and as a potential therapeutic target for eliminating that most adventitious cell [47].

### Eph/Ephrin and chondrosarcoma

Chondrosarcoma is a chondrogenic malignant tumor, accounting for approximately 20% of primary malignant tumors of bone, usually occurring in the pelvis or long bones, and often showing mucinous changes, calcification and ossification. Chondrosarcoma can be solitary or can arise from the malignant transformation of endogenous chondrosarcoma and osteochondroma [48]. Kalinski et al. [49] found that Ephrin-A5 was downregulated in chondrosarcoma, and that the aim was negatively correlated with the malignancy of chondrosarcoma. Therefore, Ephrin-A5 may function as an oncogene in chondrosarcoma, and Ephrin-A5 is associated with cell adhesion, and it is hypothesized that the downregulation of Ephrin-A5 leads to the upregulation of tumor cell migration and invasion ability through cell adhesion function, which of course needs to be further explored. A significant correlation was found that EphA2 with worse overall survival of dedifferentiated chondrosarcoma [37].

## Eph/Ephrin and bone cancer pain (bcp)

Treating bone cancer pain continues to be a clinical challenge and underlying mechanisms of bone cancer pain remain elusive. The metastases of malignant tumors often occur, and bone is one of the most common metastatic organs of tumors, which can cause osteogenic or osteolytic changes in bone. According to the study, 60% to 84% of advanced cancer patients will experience bone pain of varying degrees [50]. When tumors metastasize, the survival cycle of patients will be significantly reduced, and serious complications such as pathological fractures, bone cancer pain (BCP), and nerve compression will occur on a daily basis. Currently, there is no effective treatment for BCP, but mainly opioid analgesics are used, so there is an urgent need to find a way to alleviate BCP. Ephrin-B1 and EphB1 expression in the spinal cord was significantly increased in a bone metastasis model of Breast cancer. BCP was induced by intra-tibial inoculation of Walker 256 mammary gland carcinoma cells. Intrathecal injection of EphB1 blocker EphB1-Fc into the mouse spinal cord significantly reduced the mechanical pain caused by bone cancer, and the expression of interleukin-1B (IL-1B), interleukin-6 (IL-6) and tumor necrosis factor-α (TNF-α) was upregulated in mice with bone metastasis model relative to normal mice, while their levels were significantly reduced after intrathecal injection of EphB1-Fc [7]. Therefore, intrathecal injection of EphB1-Fc may be an effective way to reduce the occurrence of BCP. Liu et al. [51] found that BCP was associated with the upregulation of EphB1 and EphrinB2 in the dorsal horn and primary neurons of the spinal cord. Specific blockade of EphB1 reduced the phosphorylation of NR1 and NR2B receptors, Src, and subsequent activation of various Ca^2+^ -dependent signaling enzymes, CREB, c-Fos, and activation of painful behaviors in the BCP. The positive cut of EphB1 also relieves the tolerance of BCP to morphine treatment, so EphB1 receptor signaling in the spinal cord is critical for the development of bone cancer pain and the relief of morphine tolerance in cancer pain. After intrathecal injection of the EphB1 receptor blocker reagent EphB2-Fc, pathological observations revealed an improvement in tumor-associated bone destruction. In addition, EphB1 receptors are also involved in BCP through Toll-like receptor activation of IL-1β and TNF-α in neuroglia and microglia in the spinal cord [52]. Thus, EphB1 is involved in the development and progression of BCP, and the day is associated with morphine tolerance and improves bone destruction in BCP patients. In other aspects, Yin Wang et al. demonstrated that EphA4 receptor is involved in the generation and maintenance of CFA-induced chronic inflammatory pain and that blocking the spinal EphA4 receptor could relieve persistent pain behaviors in mice [53].

## CONCLUSIONS

**Figure 2:**
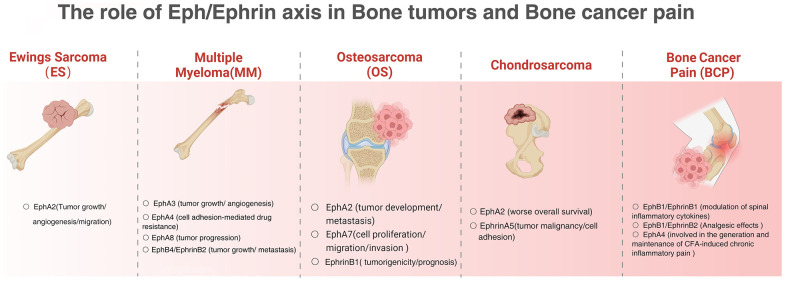
The role of Eph/Ephrin axis in bone tumors and bone cancer pain.

Eph/Ephrin has emerged as a regulator of many cancers, capable of enhancing or inhibiting the activity of oncogenic signals. However, little is known about how Eph/Ephrin regulates tumor progression at the molecular level. Eph/Ephrin is involved in tumor angiogenesis, chemoresistance and BCP development, and may become a marker for tumor grading and prognosis, and is expected to improve tumor-related bone destruction. Specific targeting agents are currently a hot topic of research and have been studied in Eph/Ephrin. Anti-EphrinA4-kacinimycin conjugates can effectively target triple negative Vulgar adenocarcinoma and ovarian tumor initiating cells, leading to sustained tumor regression. Activation of AKT signaling by the EPHA2/ephrin-A1-CAV1 axis to secrete bFGF and has been suggested as a potential target for chemotherapeutic agents [44]. The EphA2 inhibitor ALW-II-41-27 has a dose-dependent antitumor effect in patient-derived *in vitro* models; as such, EphA2 targeting represents a promising new therapeutic strategy for targeting osteosarcoma [37]. In conclusion, Eph/Ephrin plays a clear role in primary or metastatic bone tumors, and a full understanding of its intrinsic molecular mechanisms of action will help to elucidate the mechanisms of bone tumorigenesis and metastasis and contribute to the development of new anti-cancer therapies (Figure 2).

## References

[1] Hirai H, Maru Y, Hagiwara K, Nishida J, Takaku F. A novel putative tyrosine kinase receptor encoded by the eph gene. Science. 1987; 238:1717–20. 10.1126/science.2825356 2825356

[2] Pergaris A, Danas E, Goutas D, Sykaras AG, Soranidis A, Theocharis S. The Clinical Impact of the EPH/Ephrin System in Cancer: Unwinding the Thread. Int J Mol Sci. 2021; 22:8412. 10.3390/ijms22168412 34445116 PMC8395090

[3] Hadjimichael AC, Pergaris A, Kaspiris A, Foukas AF, Kokkali S, Tsourouflis G, Theocharis S. The EPH/Ephrin System in Bone and Soft Tissue Sarcomas’ Pathogenesis and Therapy: New Advancements and a Literature Review. Int J Mol Sci. 2022; 23:5171. 10.3390/ijms23095171 35563562 PMC9100911

[4] Baek JM, Cheon YH, Kwak SC, Jun HY, Yoon KH, Lee MS, Kim JY. Claudin 11 regulates bone homeostasis via bidirectional EphB4-EphrinB2 signaling. Exp Mol Med. 2018; 50:1–18. 10.1038/s12276-018-0076-3 29700355 PMC5938033

[5] Liao C, Cheng T, Wang S, Zhang C, Jin L, Yang Y. Shear stress inhibits IL-17A-mediated induction of osteoclastogenesis via osteocyte pathways. Bone. 2017; 101:10–20. 10.1016/j.bone.2017.04.003 28414140

[6] Wu M, Ai W, Chen L, Zhao S, Liu E. Bradykinin receptors and EphB2/EphrinB2 pathway in response to high glucose-induced osteoblast dysfunction and hyperglycemia-induced bone deterioration in mice. Int J Mol Med. 2016; 37:565–74. 10.3892/ijmm.2016.2457 26782642 PMC4771119

[7] Dong Y, Mao-Ying QL, Chen JW, Yang CJ, Wang YQ, Tan ZM. Involvement of EphB1 receptor/ephrinB1 ligand in bone cancer pain. Neurosci Lett. 2011; 496:163–7. 10.1016/j.neulet.2011.04.008 21514363

[8] Wijeratne DT, Rodger J, Wood FM, Fear MW. The role of Eph receptors and Ephrins in the skin. Int J Dermatol. 2016; 55:3–10. 10.1111/ijd.12968 26498559

[9] Larsen AB, Pedersen MW, Stockhausen MT, Grandal MV, van Deurs B, Poulsen HS. Activation of the EGFR gene target EphA2 inhibits epidermal growth factor-induced cancer cell motility. Mol Cancer Res. 2007; 5:283–93. 10.1158/1541-7786.MCR-06-0321 17374733

[10] Prydz K, Halstensen TS, Holen HL, Aasheim HC. Ephrin-B3 binds both cell-associated and secreted proteoglycans. Biochem Biophys Res Commun. 2018; 503:2212–7. 10.1016/j.bbrc.2018.06.140 29953858

[11] Irie F, Okuno M, Matsumoto K, Pasquale EB, Yamaguchi Y. Heparan sulfate regulates ephrin-A3/EphA receptor signaling. Proc Natl Acad Sci USA. 2008; 105:12307–12. 10.1073/pnas.0801302105 18715996 PMC2527907

[12] Kou CJ, Kandpal RP. Differential Expression Patterns of Eph Receptors and Ephrin Ligands in Human Cancers. Biomed Res Int. 2018; 2018:7390104. 10.1155/2018/7390104 29682554 PMC5851329

[13] Pasquale EB. Eph receptors and ephrins in cancer: bidirectional signalling and beyond. Nat Rev Cancer. 2010; 10:165–80. 10.1038/nrc2806 20179713 PMC2921274

[14] Pasquale EB. Eph receptor signalling casts a wide net on cell behaviour. Nat Rev Mol Cell Biol. 2005; 6:462–75. 10.1038/nrm1662 15928710

[15] Pasquale EB. Eph-ephrin bidirectional signaling in physiology and disease. Cell. 2008; 133:38–52. 10.1016/j.cell.2008.03.011 18394988

[16] Genander M, Frisén J. Ephrins and Eph receptors in stem cells and cancer. Curr Opin Cell Biol. 2010; 22:611–6. 10.1016/j.ceb.2010.08.005 20810264

[17] Castaño J, Davalos V, Schwartz S Jr, Arango D. EPH receptors in cancer. Histol Histopathol. 2008; 23:1011–23. 10.14670/HH-23.1011 18498077

[18] Banerjee SL, Lessard F, Chartier FJM, Jacquet K, Osornio-Hernandez AI, Teyssier V, Ghani K, Lavoie N, Lavoie JN, Caruso M, Laprise P, Elowe S, Lambert JP, Bisson N. EPH receptor tyrosine kinases phosphorylate the PAR-3 scaffold protein to modulate downstream signaling networks. Cell Rep. 2022; 40:111031. 10.1016/j.celrep.2022.111031 35793621

[19] Sato S, Vasaikar S, Eskaros A, Kim Y, Lewis JS, Zhang B, Zijlstra A, Weaver AM. EPHB2 carried on small extracellular vesicles induces tumor angiogenesis via activation of ephrin reverse signaling. JCI Insight. 2019; 4:e132447. 10.1172/jci.insight.132447 31661464 PMC6962030

[20] Tanaka M, Sasaki K, Kamata R, Sakai R. The C-terminus of ephrin-B1 regulates metalloproteinase secretion and invasion of cancer cells. J Cell Sci. 2007; 120:2179–89. 10.1242/jcs.008607 17567680

[21] Huang GH, Guo L, Zhu L, Liu XD, Sun ZL, Li HJ, Xu NJ, Feng DF. Neuronal GAP-Porf-2 transduces EphB1 signaling to brake axon growth. Cell Mol Life Sci. 2018; 75:4207–22. 10.1007/s00018-018-2858-0 29938386 PMC11105709

[22] Kulig P, Milczarek S, Bakinowska E, Szalewska L, Baumert B, Machaliński B. Lenalidomide in Multiple Myeloma: Review of Resistance Mechanisms, Current Treatment Strategies and Future Perspectives. Cancers (Basel). 2023; 15:963. 10.3390/cancers15030963 36765919 PMC9913106

[23] Zhao C, Irie N, Takada Y, Shimoda K, Miyamoto T, Nishiwaki T, Suda T, Matsuo K. Bidirectional ephrinB2-EphB4 signaling controls bone homeostasis. Cell Metab. 2006; 4:111–21. 10.1016/j.cmet.2006.05.012 16890539

[24] Pennisi A, Ling W, Li X, Khan S, Shaughnessy JD Jr, Barlogie B, Yaccoby S. The ephrinB2/EphB4 axis is dysregulated in osteoprogenitors from myeloma patients and its activation affects myeloma bone disease and tumor growth. Blood. 2009; 114:1803–12. 10.1182/blood-2009-01-201954 19597185 PMC2738568

[25] Oshima T, Abe M, Asano J, Hara T, Kitazoe K, Sekimoto E, Tanaka Y, Shibata H, Hashimoto T, Ozaki S, Kido S, Inoue D, Matsumoto T. Myeloma cells suppress bone formation by secreting a soluble Wnt inhibitor, sFRP-2. Blood. 2005; 106:3160–5. 10.1182/blood-2004-12-4940 16030194

[26] Guan M, Liu L, Zhao X, Wu Q, Yu B, Shao Y, Yang H, Fu X, Wan J, Zhang W. Copy number variations of EphA3 are associated with multiple types of hematologic malignancies. Clin Lymphoma Myeloma Leuk. 2011; 11:50–3. 10.3816/CLML.2011.n.006 21454190

[27] La Rocca F, Airoldi I, Di Carlo E, Marotta P, Falco G, Simeon V, Laurenzana I, Trino S, De Luca L, Todoerti K, Villani O, Lackmann M, D’Auria F, et al. EphA3 targeting reduces *in vitro* adhesion and invasion and *in vivo* growth and angiogenesis of multiple myeloma cells. Cell Oncol (Dordr). 2017; 40:483–96. 10.1007/s13402-017-0338-4 28721629 PMC13001579

[28] Ding L, Shen Y, Ni J, Ou Y, Ou Y, Liu H. EphA4 promotes cell proliferation and cell adhesion-mediated drug resistance via the AKT pathway in multiple myeloma. Tumour Biol. 2017; 39:1010428317694298. 10.1177/1010428317694298 28351297

[29] Peng Y, Song X, Lan J, Wang X, Wang M. Bone marrow stromal cells derived exosomal miR-10a and miR-16 may be involved in progression of patients with multiple myeloma by regulating EPHA8 or IGF1R/CCND1. Medicine (Baltimore). 2021; 100:e23447. 10.1097/MD.0000000000023447 33530159 PMC7850735

[30] Posthumadeboer J, Piersma SR, Pham TV, van Egmond PW, Knol JC, Cleton-Jansen AM, van Geer MA, van Beusechem VW, Kaspers GJ, van Royen BJ, Jiménez CR, Helder MN. Surface proteomic analysis of osteosarcoma identifies EPHA2 as receptor for targeted drug delivery. Br J Cancer. 2013; 109:2142–54. 10.1038/bjc.2013.578 24064975 PMC3798973

[31] Fritsche-Guenther R, Noske A, Ungethüm U, Kuban RJ, Schlag PM, Tunn PU, Karle J, Krenn V, Dietel M, Sers C. De novo expression of EphA2 in osteosarcoma modulates activation of the mitogenic signalling pathway. Histopathology. 2010; 57:836–50. 10.1111/j.1365-2559.2010.03713.x 21166698

[32] Haghiralsadat F, Amoabediny G, Naderinezhad S, Nazmi K, De Boer JP, Zandieh-Doulabi B, Forouzanfar T, Helder MN. EphA2 Targeted Doxorubicin-Nanoliposomes for Osteosarcoma Treatment. Pharm Res. 2017; 34:2891–900. 10.1007/s11095-017-2272-6 29110283

[33] Hsu K, Middlemiss S, Saletta F, Gottschalk S, McCowage GB, Kramer B. Chimeric Antigen Receptor-modified T cells targeting EphA2 for the immunotherapy of paediatric bone tumours. Cancer Gene Ther. 2021; 28:321–34. 10.1038/s41417-020-00221-4 32873870 PMC8057949

[34] Chiabotto G, Grignani G, Todorovic M, Martin V, Centomo ML, Prola E, Giordano G, Merlini A, Miglio U, Berrino E, Napione L, Isella C, Capozzi F, et al. Pazopanib and Trametinib as a Synergistic Strategy against Osteosarcoma: Preclinical Activity and Molecular Insights. Cancers (Basel). 2020; 12:1519. 10.3390/cancers12061519 32531992 PMC7352822

[35] Song W, Hwang Y, Youngblood VM, Cook RS, Balko JM, Chen J, Brantley-Sieders DM. Targeting EphA2 impairs cell cycle progression and growth of basal-like/triple-negative breast cancers. Oncogene. 2017; 36:5620–30. 10.1038/onc.2017.170 28581527 PMC5629103

[36] Peng Q, Chen L, Wu W, Wang J, Zheng X, Chen Z, Jiang Q, Han J, Wei L, Wang L, Huang J, Ma J. EPH receptor A2 governs a feedback loop that activates Wnt/β-catenin signaling in gastric cancer. Cell Death Dis. 2018; 9:1146. 10.1038/s41419-018-1164-y 30451837 PMC6242896

[37] Giordano G, Merlini A, Ferrero G, Mesiano G, Fiorino E, Brusco S, Centomo ML, Leuci V, D’Ambrosio L, Aglietta M, Sangiolo D, Grignani G, Pignochino Y. EphA2 Expression in Bone Sarcomas: Bioinformatic Analyses and Preclinical Characterization in Patient-Derived Models of Osteosarcoma, Ewing’s Sarcoma and Chondrosarcoma. Cells. 2021; 10:2893. 10.3390/cells10112893 34831119 PMC8616526

[38] Wu X, Yan L, Liu Y, Xian W, Wang L, Ding X. MicroRNA-448 suppresses osteosarcoma cell proliferation and invasion through targeting EPHA7. PLoS One. 2017; 12:e0175553. 10.1371/journal.pone.0175553 28604772 PMC5467824

[39] Tu Y, Cai Q, Zhu X, Xu M. Down-regulation of HCP5 inhibits cell proliferation, migration, and invasion through regulating EPHA7 by competitively binding miR-101 in osteosarcoma. Braz J Med Biol Res. 2021; 54:e9161. 10.1590/1414-431X20209161 33439936 PMC7798137

[40] Varelias A, Koblar SA, Cowled PA, Carter CD, Clayer M. Human osteosarcoma expresses specific ephrin profiles: implications for tumorigenicity and prognosis. Cancer. 2002; 95:862–9. 10.1002/cncr.10749 12209731

[41] Abdou AG, Abd El-Wahed MM, Asaad NY, Samaka RM, Abdallaha R. Immunohistochemical profile of ephrin A4 expression in human osteosarcoma. APMIS. 2009; 117:277–85. 10.1111/j.1600-0463.2009.02448.x 19338516

[42] Abdou AG, Abd el-Wahed MM, Asaad NY, Samaka RM, Abdallaha R. Ephrin A4 expression in osteosarcoma, impact on prognosis, and patient outcome. Indian J Cancer. 2010; 47:46–52. 10.4103/0019-509X.58859 20071790

[43] Evdokimova V, Gassmann H, Radvanyi L, Burdach SE. Current State of Immunotherapy and Mechanisms of Immune Evasion in Ewing Sarcoma and Osteosarcoma. Cancers (Basel). 2022; 15:272. 10.3390/cancers15010272 36612267 PMC9818129

[44] Sáinz-Jaspeado M, Huertas-Martinez J, Lagares-Tena L, Martin Liberal J, Mateo-Lozano S, de Alava E, de Torres C, Mora J, Del Muro XG, Tirado OM. EphA2-induced angiogenesis in ewing sarcoma cells works through bFGF production and is dependent on caveolin-1. PLoS One. 2013; 8:e71449. 10.1371/journal.pone.0071449 23951165 PMC3741133

[45] van der Schaft DW, Hillen F, Pauwels P, Kirschmann DA, Castermans K, Egbrink MG, Tran MG, Sciot R, Hauben E, Hogendoorn PC, Delattre O, Maxwell PH, Hendrix MJ, Griffioen AW. Tumor cell plasticity in Ewing sarcoma, an alternative circulatory system stimulated by hypoxia. Cancer Res. 2005; 65:11520–8. 10.1158/0008-5472.CAN-05-2468 16357161

[46] Garcia-Monclús S, López-Alemany R, Almacellas-Rabaiget O, Herrero-Martín D, Huertas-Martinez J, Lagares-Tena L, Alba-Pavón P, Hontecillas-Prieto L, Mora J, de Álava E, Rello-Varona S, Giangrande PH, Tirado OM. EphA2 receptor is a key player in the metastatic onset of Ewing sarcoma. Int J Cancer. 2018; 143:1188–201. 10.1002/ijc.31405 29582409 PMC6103826

[47] Keskin T, Rucci B, Cornaz-Buros S, Martin P, Fusco C, Broye L, Cisarova K, Perez EM, Letovanec I, La Rosa S, Cherix S, Diezi M, Renella R, et al. A live single-cell reporter assay links intratumor heterogeneity to metastatic proclivity in Ewing sarcoma. Sci Adv. 2021; 7:eabf9394. 10.1126/sciadv.abf9394 34215585 PMC11060044

[48] Puls F, Niblett AJ, Mangham DC. Molecular pathology of bone tumours: diagnostic implications. Histopathology. 2014; 64:461–76. 10.1111/his.12275 24428620

[49] Kalinski T, Röpke A, Sel S, Kouznetsova I, Röpke M, Roessner A. Down-regulation of ephrin-A5, a gene product of normal cartilage, in chondrosarcoma. Hum Pathol. 2009; 40:1679–85. 10.1016/j.humpath.2009.03.024 19695673

[50] Zajączkowska R, Kocot-Kępska M, Leppert W, Wordliczek J. Bone Pain in Cancer Patients: Mechanisms and Current Treatment. Int J Mol Sci. 2019; 20:6047. 10.3390/ijms20236047 31801267 PMC6928918

[51] Liu S, Liu WT, Liu YP, Dong HL, Henkemeyer M, Xiong LZ, Song XJ. Blocking EphB1 receptor forward signaling in spinal cord relieves bone cancer pain and rescues analgesic effect of morphine treatment in rodents. Cancer Res. 2011; 71:4392–402. 10.1158/0008-5472.CAN-10-3870 21555368

[52] Liu S, Liu YP, Song WB, Song XJ. EphrinB-EphB receptor signaling contributes to bone cancer pain via Toll-like receptor and proinflammatory cytokines in rat spinal cord. Pain. 2013; 154:2823–35. 10.1016/j.pain.2013.08.017 23973554

[53] Wang Y, Wen C, Xie G, Jiang L. Blockade of Spinal EphA4 Reduces Chronic Inflammatory Pain in Mice. Neurol Res. 2021; 43:528–34. 10.1080/01616412.2021.1884798 33541257

